# Oscillatory Brain Dynamics during Sentence Reading: A Fixation-Related Spectral Perturbation Analysis

**DOI:** 10.3389/fnhum.2016.00191

**Published:** 2016-04-29

**Authors:** Lorenzo Vignali, Nicole A. Himmelstoss, Stefan Hawelka, Fabio Richlan, Florian Hutzler

**Affiliations:** Centre for Cognitive Neuroscience, University of SalzburgSalzburg, Austria

**Keywords:** brain oscillations, electroencephalography, sentence processing, eye movements, semantic violation

## Abstract

The present study investigated oscillatory brain dynamics during self-paced sentence-level processing. Participants read fully correct sentences, sentences containing a semantic violation and “sentences” in which the order of the words was randomized. At the target word level, fixations on semantically unrelated words elicited a lower-beta band (13–18 Hz) desynchronization. At the sentence level, gamma power (31–55 Hz) increased linearly for syntactically correct sentences, but not when the order of the words was randomized. In the 300–900 ms time window after sentence onsets, theta power (4–7 Hz) was greater for syntactically correct sentences as compared to sentences where no syntactic structure was preserved (random words condition). We interpret our results as conforming with a recently formulated predictive-coding framework for oscillatory neural dynamics during sentence-level language comprehension. Additionally, we discuss how our results relate to previous findings with serial visual presentation vs. self-paced reading.

## Introduction

Recent accounts of visual word recognition describe the reading process as guided by an interplay between bottom-up incoming information and forward inferences (or predictions; Hagoort, 2005, 2013, 2014; Price and Devlin, 2011). While we read, incoming linguistic inputs are sequentially integrated into the language context (e.g., a sentence, a text or a discourse) at a rate which, in silent reading, is on the verge of 350 words per minute (e.g., Rayner, 1998). Recent findings indicate that fast readers employ highly efficient reading strategies by complementing bottom-up incoming information with predictions concerning probable upcoming words (DeLong et al., 2005; Hawelka et al., 2015). Sentence level predictions could be inferred based on previous knowledge or on context-based semantic information (Altmann and Kamide, 1999; Kamide et al., 2003).

The fact that predictions play a role during sentence-level processing is undisputed. Evidence stems, for example, from eye movement (EM) studies which assessed anticipatory looking, skipping probabilities and fixation durations—variables which are consistently affected by predictability measures (e.g., cloze probability—Balota et al., 1985; Kliegl et al., 2006; Rayner, 2009; Risse et al., 2014; Hawelka et al., 2015). Further support for context based predictions stems from electroencephalographical (EEG) measures of the reading process. Event related brain potentials (ERPs), such as the well documented N400 ERP component (for reviews see Kutas and Federmeier, 2011), were shown to reflect the degree of anomaly (“integration view”) as well as the predictability (“prediction view”) of an incoming word (for a review see Federmeier, 2007). DeLong et al. (2005) investigated the modulation of the N400 as related to expected vs. unexpected article (“a” and “an”)/noun pairs. Results showed that the size of the N400 was larger for articles and nouns that mismatched the sentence-context prediction (e.g., “an airplane” when “a kite” was the most probable continuation). Furthermore, the N400’s mean amplitudes were inversely correlated with the cloze probability of both, the articles and the nouns. These findings suggest that context-based predictions are rapidly and continuously adjusted in correspondence to incoming inputs. In line with this hypothesis, numerous magnetoencephalography (MEG) studies (Pammer et al., 2004; Cornelissen et al., 2009; Wheat et al., 2010; Dikker and Pylkkanen, 2011; Woodhead et al., 2014) as well as EEG studies (Dambacher et al., 2009) reported early effects (within 220 ms) of top-down predictions on visual word recognition. Accordingly, several neurobiological models of language comprehension embraced this predictive coding perspective (Hagoort, 2005, 2013; Jung-Beeman, 2005; Lau et al., 2008). Although partially distinct from each other, these models share two main assumptions: (1) the reading network encompasses brain areas mainly distributed across the left hemisphere; and (2) these areas interact with each other through top-down and bottom-up connections. For the purpose of the present study we will not focus on the functional neuroanatomy of the reading network (for a review see Price, 2012; Taylor et al., 2013; Martin et al., 2015), but rather on neuronal communication between and within areas as related to sentence-level predictions.

A growing body of evidence suggests that oscillatory dynamics represent a feasible mean of inter- and intra-areal communication (Gray et al., 1989; Bressler, 1995). More specifically, synchronous oscillatory activity was evidenced as a possible mechanism for the propagation of top-down and bottom-up information across cortical levels (von Stein et al., 2000; Engel et al., 2001; Varela et al., 2001; Bar et al., 2006; Hipp et al., 2011; Singer, 2011; Bressler and Richter, 2015). Accordingly, language related functions were repeatedly associated to changes in power and coherence measures in the theta (4–7 Hz), alpha (8–12 Hz), beta (13–30 Hz) and gamma (above 30 Hz) band (for reviews see Bastiaansen and Hagoort, 2006; Lewis et al., 2015). In a recent proposal Lewis and Bastiaansen (2015) suggested a strong link between oscillatory brain activity and forward inferences generated during sentence-level language comprehension. In this proposal, the authors attempted to consolidate previous language-related literature (for a review see Lewis et al., 2015) into a unified predictive coding framework for fast oscillatory neural dynamics. In this framework, two frequency bands are particularly important: the beta band (13–30 Hz) and the gamma band (35–75 Hz). According to the authors, sentence processing entails the formation of a functional network or NeuroCognitive Network (NCN—self-organized, large scale distributed cortical network) encompassing most language related areas. The maintenance or change of state in this network is supposed to be reflected in beta band oscillatory activity. More specifically, when the NCN is under revision/change (e.g., a semantically unrelated word is encountered), one would expect a beta desynchronization as compared to a synchronization for fully correct sentences. Functionally distinct, but strongly related, is the role proposed for gamma band activity. Gamma activity is expected to reflect the congruity between incoming linguistic inputs and pre-activated predictions. Gamma synchronization would reflect the successful matching of top-down predictions with bottom-up incoming information whereas no increase in gamma power (or even a desynchronization) is to be expected in case of a mismatch. This interpretation was based on Herrmann et al. (2004) “match-and-utilization” proposal as well as on recent proposals for language processing (Peña and Melloni, 2012; Wang et al., 2012b).

Electrophysiological correlates of sentence reading were, up to now, mainly recorded using “serial visual presentation” (SVP) paradigms. Thus, the wealth of evidence considered by Lewis and Bastiaansen (2015) for their framework stemmed from SVP. In the SVP approach a sentence is broken down into a series of individual words presented on the screen in isolation and separated by relatively long interstimulus intervals (ITI—usually about 500 ms). Single word presentation renders unnecessary sequential saccades to bring new stimuli to the foveal point of vision (thus avoiding horizontal EMs resulting in ocular artifacts, Henderson et al., 2013). Furthermore, long ITIs prevent the temporal overlap of the processing of successive stimuli, and thus the overlap of (and the necessity to disentangle) the corresponding brain potentials (Sereno and Rayner, 2003; Kliegl et al., 2012). SVPs were proven to be a reliable tool for the study of visual word recognition (Kutas and Federmeier, 2007). However, serial presentation might be inadequate when it comes to the study of sentence level reading. Serial presentation may distort, for instance, the normal time-course of syntactic and semantic integration and the processes of disambiguation (in case of erroneous or imprecise syntactic or semantic parsing). Whereas the former is likely to be altered by a superimposed timeline of word recognition (duration of stimulus presentation plus inter trial interval), the latter is fully inhibited as no regressive saccades can be performed to previously encountered words (Rayner, 1998; Pernet et al., 2007; Kliegl et al., 2012; Schotter et al., 2014).

For studying the reading process, fixation-related potentials (FRPs) are an alternative to the SVP paradigms (Hutzler et al., 2007; Dimigen et al., 2011, 2012). FRPs are based on the coregistration of EM and EEG data. In the FRP approach (contrary to classical ERPs), the onset of a cognitive process is not defined by an externally triggered event (i.e., stimulus onset), but it is indicated by EMs (i.e., the first fixation on a word). This allows participants to read at their own pace (self-paced reading), thus providing a higher ecological validity of the reading process and the corresponding brain signal.

In the context of neuroimaging, inconsistent findings among studies have been attributed to methodological aspects such as task demands and “unnatural” stimulus presentation (too long presentation durations; see Dehaene and Cohen, 2011; Schuster et al., 2015). Such methodological characteristics—which could induce an elevated level of processing and, ultimately, alter brain responses—can largely be circumvented by means of administering more ecologically valid approaches such those made possible by the FRP paradigm. In the context of electrophysiology, previous FRPs experiments showed how this approach permits a deeper understanding of the relationship between EM and brain signals (Dimigen et al., 2012). To illustrate, in self-paced reading the timing of word recognition can be more directly inferred (Kliegl et al., 2012). Accordingly, results from SVP and FRPs paradigms differ in temporal aspects (e.g., Hutzler et al., 2013) as well as concerning the specific cognitive processes involved. Whereas temporal aspects were proven to affect the timing of signal change (see Dimigen et al., 2011; Hutzler et al., 2013), differences in cognitive processing are most likely to affect inter- and intra-areal communication and, as a consequence, the observed oscillatory activity (for a review see Bressler, 1995).

Using the FRP paradigm, Metzner et al. (2015) re-investigated Hagoort et al.’s (2004) SVP-based results. Although being able to replicate classical ERP findings (i.e., N400), Metzner et al. (2015) reported qualitative differences in oscillatory brain dynamics for FRPs compared to serial presentation. More specifically, the authors reported an increase in power in the delta range and a decrease in power in the upper alpha range following the fixation on a word incongruent with common world knowledge (e.g., *Rome is the capital of France*). This result is at odds not only with SVP findings as reported by Hagoort et al. (2004), but also with predictions concerning oscillatory neural dynamics as described by Lewis and Bastiaansen’s (2015) framework. Metzner et al.’s (2015) findings, although only evidence from a single study, cast general doubts concerning the ecological validity of oscillatory dynamics stemming from SVP. As aforementioned, the rather artificial settings imposed by SVP paradigms might not be generalizable to natural reading. As an alternative explanation, the methodological approach adopted by Metzner et al. (2015) was not optimally suited to test predictions made by Lewis and Bastiaansen’s (2015) framework. To illustrate, in Metzner et al. (2015) the target word was the sentence final word in 90 out of 120 sentences, whereas this was never the case in Hagoort et al.’s (2004) stimulus material. Furthermore, differences in the experimental design as well as in the analysis (FFT vs. Wavelets) could be plausible explanations for Metzner et al.’s (2015) divergent findings.

The aim of the present study was to use an ecologically valid fixation-related brain potentials paradigm to test predictions of Lewis and Bastiaansen’s (2015) framework. To do so, we kept the experimental paradigm and methodological approach as similar as possible to previous SVP studies. More specifically, we used an adaptation of Bastiaansen et al.’s (2010) paradigm including fully correct sentences, sentences embedding a semantic violation, and two conditions where the order of the words was randomized (no syntactic structure left). According to the framework of Lewis and Bastiaansen (2015), we expected semantically unrelated words to cause a beta desynchronization and a decrease in gamma power. Similar to Bastiaansen et al. (2010) we also investigated power changes related to sentence-level processing. We traced the development of oscillatory dynamics related to the unfolding of the sentence. Furthermore, in accordance with Lewis and Bastiaansen’s (2015) framework, we expected a larger gamma and beta band power when subjects read syntactically correct sentences as compared to the condition where subjects read randomly arranged words.

## Materials and Methods

### Participants

Thirty-two native German speaking students (10 males, *M* = 23.2 years, *SD* = 2.3 years) participated in this study. All participants were right–handed; had normal or corrected-to-normal vision and no history of neurological or psychiatric disorders. All participants undertook a short reading test to ensure reading proficiency (reading rate above 150 words per minute). Before testing participants gave their written informed consent. The experiment was conducted in accordance with the Declaration of Helsinki and was approved by the local ethics committee of the University of Salzburg.

### Materials and Stimuli

The stimulus material consisted of 120 quadruples of sentences. A sentence quadruple comprised two syntactically correct sentences (ORD) and two random word order sentences (RDM). The ORD conditions consisted of: (i) a fully correct sentence (ORD_COR); and (ii) a version of the same sentence which contained a semantic violation (i.e., a semantically unrelated word; ORD_SEM—see Table 1). The two RDM conditions were created by pseudo-randomly arranging the words contained in the two ORD conditions (i.e., RDM_COR and RDM_SEM—see Table 1). In the randomization process we ensured that no syntactic structure was left, but we kept the target word at the original position. The average sentence length was 9.56 words (*SD* = 1.61) and the average position of the target word in the sentence was 7.1 (*SD* = 1.37). Target words were never the initial or the final word of a sentence. Moreover, the target words were matched across conditions (i.e., for the factor COR and SEM) for the following criteria: lemma word *log*-frequency (COR: *M* = 1.38, *SD* = 0.83; SEM: *M* = 1.23, *SD* = 0.77), length (COR: *M* = 6.63, *SD* = 2.09; SEM: *M* = 6.21, *SD* = 2.00) and number of syllables (COR: *M* = 2.09, *SD* = 0.85; SEM: *M* = 2.01, *SD* = 0.94). We splitted and pseudorandomized the items in two versions of the experiment. This procedure was applied in order to ensure that each participant read only one ORD version and the opposite RDM version of each sentence quadruplet. To illustrate, a participant received the ORD-COR/RDM-SEM pairing of a particular sentence; whereas another received the ORD-SEM/RDM-COR pairing. Participants were randomly assigned to one of the two versions of the experiment (16 participants per group).

**Table 1:** **Example stimuli for each condition**.

ORD_COR	Der Rhein mündet in die **Nordsee** in der Nähe von Rotterdam.
	*(The Rhine flows into the North_Sea in the nearness of Rotterdam.)*
ORD_SEM	Der Rhein mündet in die **Konsole** in der Nähe von Rotterdam.
	*(The Rhine flows into the console in the nearness of Rotterdam.)*
RDM_COR	Der Rotterdam die Nähe in **Nordsee** mündet der in Rhein von.
	*(The Rotterdam the nearness in North_Sea flows the in Rhine of.)*
RDM_SEM	Der Rotterdam die Nähe in **Konsole** mündet der in Rhein von.
	*(The Rotterdam the nearness in console flows the in Rhine of.)*

*Note. Target word in bold*.

### Experimental Procedure

Participants were seated in a dimly lit room, at a distance of 50 cm from a 21-in (53.34 cm) CRT monitor (1024 × 768 pixel resolution, 120 Hz refresh rate). After the instruction screen, a three dots calibration routine was performed. Each trial sequence started with a fixation cross presented at the leftmost side of the screen for a maximum of 3 s. To trigger the stimulus appearance, participants had to maintain their gaze on the fixation cross for a minimum of 200 ms. If no fixation was detected, a new calibration routine was initiated. Stimuli were presented as whole sentences, in a mono-spaced bold font (Courier New, 14 pt). Participants were instructed to silently read the stimuli. The trials’ end was prompted by fixating a cross presented at the bottom right corner of the screen. A blank screen presented for a period ranging from 750 to 1220 ms served as intertrial interval. The experiment was divided in three blocks (80 trials each). At the end of each block participants took a short break (3–5 min). Each session lasted approximately 2 h.

### Recording and Analysis

#### Apparatus

Electrophysiological measures were acquired with 64 active electrodes EEG system (actiCAP, Brain Products GmbH, Germany) positioned according to the standard 10–10 system. Signals were amplified using an ActiCHamp Amplifier (Brain Products GmbH, Germany). All impedances were kept below 10 kΩ. Bipolar horizontal EOG were recorded between electrodes at the outer left and right canthus. EMs were recorded monocularly from the right eye with a SR Research Eyelink 1000 desktop mount system (SR Research, ON, Canada). The head was stabilized via a chin rest. Both the eye tracker’s and the EEG’s sampling rates were set to 500 Hz. At the beginning of each block the eye tracker was calibrated with a horizontal 3-points calibration routine. The calibration was considered as successful if the average tracking error was below 0.5° of visual angle. The calibration routine was repeated every time the fixation control at the beginning of a trial (see “Experimental procedure” Section) failed. Stimulus presentation was controlled by the Experiment Builder software (SR Research Ltd., Canada).

#### Eye Movements

For the EMs analysis we excluded all trials where the target word was skipped. We further aggregated the remaining epochs into standard EMs measures: first fixation duration (FFD), gaze duration (GD), and total viewing time (TVT). FFD is the duration of the first fixation on a word during first-pass reading. GD is the sum of the duration of all fixations on a word during first-pass reading. TVT is the sum of the duration of all (progressive and regressive) fixations on a word (i.e., first plus second-pass reading). We excluded fixations shorter than 80 ms and longer than 3 SD from the individual mean (total data loss: 2.06% of all fixations). EM measures were log-transformed and analyzed with the package *ez* (Lawrence, 2011) in *R*.

#### Time-Frequency Analysis

EEG data were preprocessed and analyzed with EEGLAB (Delorme and Makeig, 2004) and ERPLAB (Lopez-Calderon and Luck, 2014) toolboxes in MATLAB (Mathworks, Natick, MA, USA). All electrodes (except EOG channels) were re-referenced against an average reference. Data was band-pass filtered between 0.1–70 Hz and a 50 Hz notch filter was applied. Ocular and muscular artifacts were corrected with Independent Component Analysis (ICA, Makeig et al., 1996, 1997) carried out separately for each subject. ICA components loading primarily in frontal sites and showing inverted polarity between the two periocular sites were defined as putative components for horizontal EMs. This procedure was conducted in accordance with standardized procedures for identification and removal of eye activity artifacts (Jung et al., 1998, 2000). For 32 participants, an average of 7.7 components were identified as putative EM components (range = 2–17, *SD* = 4.4). Time-frequency (TF) analysis was based on Event-Related Spectral Perturbation (ERSP) as implemented in EEGLAB (Delorme and Makeig, 2004). However, for the fixation-related analysis we will not use the acronym ERSP, instead we will adopt the nomenclature Fixation-Related Spectral Perturbation (FRSP). FRSPs represent the spectral power change of a post-fixation interval as compared to a pre-fixation baseline. In line with classical ERSPs, this measure is calculated as the log ratio of the two intervals and reported in decibel (db) units. We analyzed frequencies ranging from 3 to 70 Hz with a sliding Hanning-tapered 3-cycle sinusoidal wavelet. Our analysis focused on five frequency bands: theta (4–7 Hz), alpha (8–12 Hz), lower beta (13–18 Hz), upper beta (19–30 Hz) and gamma (31–55 Hz). Power change values were averaged into six clusters of five electrodes each, uniformly distributed across the two hemispheres: frontal left (F1, F3, FC1, FC3, FC5), frontal right (F2, F4, FC2, FC4, FC6), central left (C1, C3, C5, CP1, CP3), central right (C2, C4, C6, CP2, CP4), parieto-occipital left (P5, PO3, P7, PO7, O1), parieto-occipital right (P6, PO4, P8, PO8, O2). Clusters of electrodes were selected in order to roughly correspond to previous publications by our research group (Hutzler et al., 2004, 2007) as well as from other research groups (Barber et al., 2004, 2011). These clusters were proven to be well suited to investigate electrophysiological correlates of the reading process by means of both FRPs (Hutzler et al., 2007) and traditional ERP measures (Barber et al., 2004, 2011; Hutzler et al., 2004).

##### Event-Related Spectral Perturbation Time-locked to the Target Word

In order to investigate effects of semantically unrelated words on the TF data, EEG recordings were segmented from −1000 to +1500 ms time-locked to the onset of the fixation on the critical word. As baseline we used the 1000 ms period before fixation onset. Because of temporal smoothing the resulting epochs ranged from −440 to 940 ms. In order to investigate effects of the target word on TF representations (TFRs) of single data trial we will mainly focus on the comparison of the two syntactically correct conditions (i.e., ORD_COR and ORD_SEM). Mean measures of power change were included into a repeated measure analysis of variance (ANOVA). Factors in the analysis included temporal window (0–300 ms, 300–600 ms), hemisphere (left and right hemisphere), electrode cluster (frontal, central and parieto-occipital clusters) and condition (ORD_CORR vs. ORD_SEM). *Post hoc* contrasts between conditions were carried out for those time windows showing a significant Cluster * Condition interaction.

##### Event-Related Spectral Perturbation Time-locked to Sentence Onset

For the sentence level analysis we time-locked each epoch to the onset of the fixation on the first word of the sentence. Each epoch included −1000 ms pre-stimulus baseline and +2500 ms post fixation onsets. Temporal smoothing generated epochs ranging from −440 to 1940 ms. In order to investigate the evolution of power changes across the sentence we merged FRSP values of the two ORD conditions and we compared them to the merged values of the RDM conditions. Contrary to the target word level analysis, we had no prior assumptions concerning the lateralization of the effects. We therefore did not include the factor Hemisphere in the sentence-level analysis. Mean measures of power change were included into an overall ANOVA with factors: time windows (0–300 ms, 300–600 ms, 600–900 ms, 900–1200 ms, 1200–1500 ms, 1500–1800 ms), electrode clusters (frontal left, frontal right, central left, central right, parieto-occipital left, parieto-occipital right) and conditions (ORD vs. RDM). *Post hoc* contrasts between conditions were carried out for those time windows showing a significant Cluster * Condition interaction. We limited our power analysis to the first 1800 ms after the fixation on the first word of the sentence. This value was chosen, because it is—on the one hand—sufficiently long so that the participants encountered the target words (in the vast majority of the cases), but it is—on the other hand—sufficiently short to avoid perturbations (as far as possible) by different reading times (due to differences in the participants’ reading rate and differences in sentence lengths).

## Results

### Eye Movement Results

EMs’ measures (i.e., FFDs, GDs and TVT) were analyzed with a repeated measure ANOVA with Condition (the four conditions) as within subject factor.

**Figure 1:**
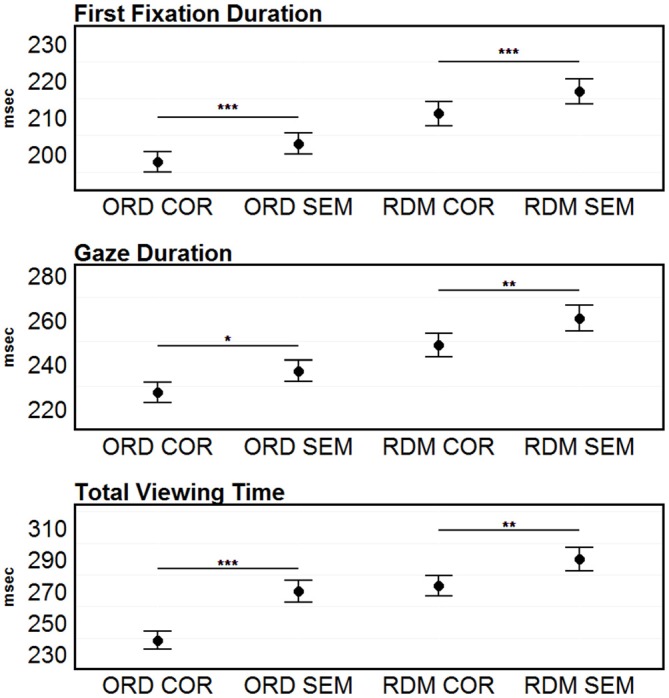
**Eye movement (EM) results.** First-fixation durations (FFDs), gaze durations (GDs) and total viewing time (TVT) on the target words. Error bars represent 95% confidence intervals. Significant differences between the correct and the semantically incorrect condition (within the ORD and COR conditions) are marked with asterisks: ***p < 0.001; **p < 0.01; *p < 0.05.

Separate ANOVAs for each dependent measure revealed highly significant effects of Condition in FFDs (*F*_(3,93)_ = 34.27, *p* < 0.001), GDs (*F*_(3,93)_ = 25.31, *p* < 0.001) and TVT (*F*_(3,93)_ = 34.02, *p* < 0.001). *Post hoc* *t*-tests revealed significantly longer FFDs (203 vs. 198, *t*_(31)_ = 2.95, *p* < 0.001), GDs (237 vs. 227, *t*_(31)_ = 2.47, *p* = 0.02) and TVT (270 vs. 239, *t*_(31)_ = 5.39, *p* < 0.001) for semantically unrelated words (see Figure 1). In the comparison between the two conditions where the order of the words was randomized (no syntactic structure left) words strings containing semantically unrelated words showed longer FFDs (217 vs. 211, *t*_(31)_ = 3.30, *p* < 0.001), GDs (260 vs. 248, *t*_(31)_ = 3.27, *p* < 0.01) and TVT (290 vs. 273, *t*_(31)_ = 3.56, *p* < 0.01). The overall pattern of results is in line with previous EM findings (e.g., Rayner et al., 2004).

### Fixation-Related Spectral Perturbation Time-locked to the Target Word

The analysis of power changes at the target word level revealed significant differences in the lower beta (13–18 Hz) band when we compared ORD_COR vs. ORD_SEM conditions in the initial 0–300 ms time window. Using the same analysis, no power differences were found in the theta (4–7 Hz), alpha (8–12 Hz), upper beta (19–30 Hz) and gamma (31–55 Hz) bands.

#### Lower Beta Band

**Figure 2:**
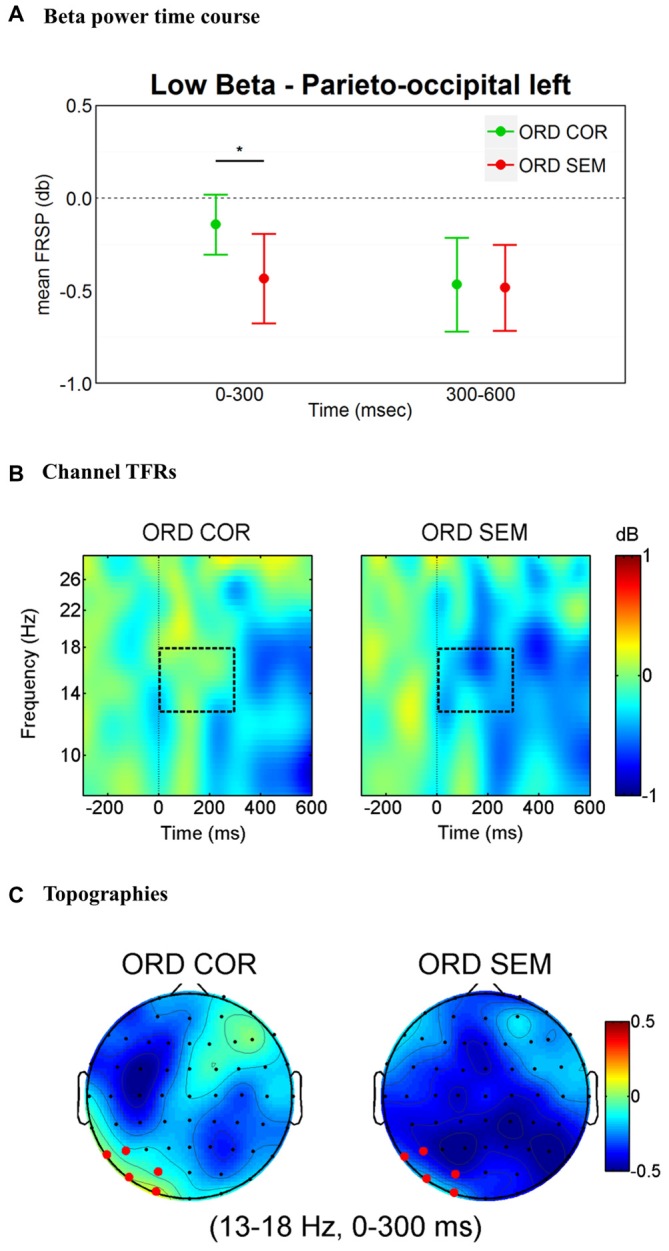
**Lower-beta band (13–18 Hz) effects time-locked to the onset of the first fixation on the target word. (A)** Left parieto-occipital cluster average lower-beta band power measures in the time windows of interest (0–300 ms and 300–600 ms). Error bars represent 95% confidence intervals. Significant differences between conditions are marked with asterisks: *p < 0.05. **(B)** Time-frequency (TF) representations of power changes in the target word level analysis. The black rectangle indicates the time and frequency range of interest at one representative channel (PO3). **(C)** Topographic maps of power change in the 0–300 ms time window. In red, the cluster of electrodes that showed a significant difference in lower-beta band activity when participants read semantically unrelated words as compared to the semantically correct target words.

The findings for the lower beta band are illustrated in Figure 2. The overall ANOVA revealed main effects of Time (*F*_(1,31)_ = 13.76, *p* = 0.001), Hemisphere (*F*_(1,31)_ = 4.46, *p* = 0.043) and Cluster (*F*_(2,62)_ = 4.29, *p* = 0.018) as well as three-way interactions Time * Hemisphere * Cluster (*F*_(2,62)_ = 6.40, *p* = 0.003) and Time * Cluster * Condition (*F*_(2,62)_ = 3.77, *p* = 0.028). A separate ANOVA for each hemisphere with factors Time (0–300 ms and 300–600 ms time windows), Cluster (three clusters) and Condition (ORD_COR vs. ORD_SEM) revealed a significant interaction Time * Cluster * Condition in the left hemisphere (*F*_(2,62)_ = 3.5, *p* = 0.036) but not in the right hemisphere (*F*_(2,62)_ = 0.55, *p* = 0.578). We followed up the left lateralized effect by performing an ANOVA for the two time windows with factors Cluster and Condition (ORD_COR vs. ORD_SEM). The 0–300 ms time window revealed a significant Cluster * Condition interaction (*F*_(2,62)_ = 4.98, *p* = 0.01). This was not the case in the 300–600 ms time window (*F*_(2,62)_ = 1.11, *p* = 0.334; see Figure 2A). Planned *t*-tests for each cluster in the 0–300 ms time window revealed a significant difference for conditions at the parieto-occipital cluster (*t*_(31)_ = 2.23, *p* = 0.033; see Figure 2C).

#### Gamma Band

The overall ANOVA revealed a significant interaction for Time * Hemisphere (*F*_(1,31)_ = 8.99, *p* = 0.005), Hemisphere * Cluster (*F*_(2,62)_ = 3.24, *p* = 0.046) and Time * Cluster * Condition (*F*_(2,62)_ = 5.68, *p* = 0.005). Separate ANOVAs for each hemisphere revealed a significant interaction Time * Cluster * Condition in the left hemisphere (*F*_(2,62)_ = 5.03, *p* = 0.009) but not in the right hemisphere (*F*_(2,62)_ = 2.11, *p* = 0.13). An ANOVA for each time window with factors Cluster (three left-lateralized clusters) and Condition (ORD_COR vs. ORD_SEM) revealed no significant Cluster * Condition interactions in both the 0–300 ms and 300–600 ms time windows (respectively: *F*_(2,62)_ = 1.62, *p* = 0.208; *F*_(2,62)_ = 0.92, *p* = 0.405).

### Fixation-Related Spectral Perturbation Time-locked to Sentence Onset

The analysis of power changes across the sentence revealed significant differences in the theta (4–7 Hz) band when we compared ORD and RDM conditions in the 300–600 and 600–900 time windows. Additionally, analysis of individual beta weights in the gamma (31–55 Hz) band showed a linear trend in the ORD but not in the RDM condition. Using the same analysis, no power differences were found for alpha (8–12 Hz), lower beta (13–18 Hz) and upper beta (19–30 Hz) bands.

#### Theta Band

**Figure 3:**
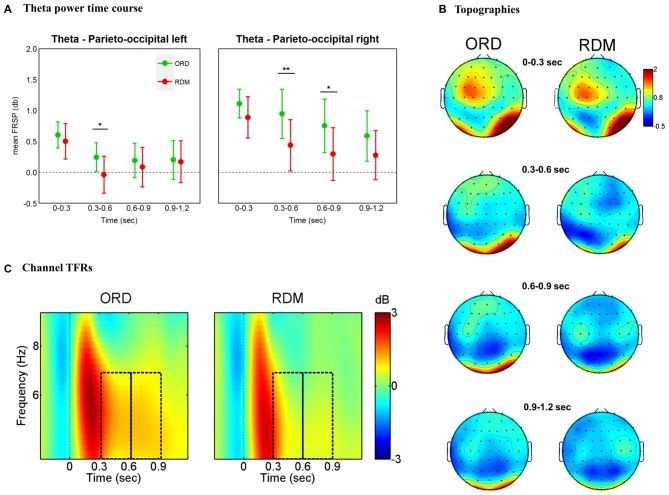
**Theta band (4–7 Hz) effects time-locked to sentence onsets. (A)** Time course of theta power for left and right parieto-occipital clusters. Mean power values are plotted in successive 300 ms time windows, up to 1200 ms. Error bars represent 95% confidence intervals. Significant differences between conditions are marked with asterisks: **p < 0.01; *p < 0.05. **(B)** Topographic maps of theta (4–7 Hz) power change in successive 300 ms time windows, up to 1200 ms. **(C)** TF representations of power changes at the sentence-level analysis. The black rectangles indicate the time windows (300–600 ms and 600–900ms) where theta power was larger for syntactically correct sentences compared to the condition where the order of the words was pseudo-randomized. Results are plotted for one representative channel (PO4).

The findings for the theta band are illustrated in Figure 3. An overall ANOVA with factors Time * Cluster * Condition revealed a main effect for Time (*F*_(5,155)_ = 15.14, *p* < 0.001), Cluster (*F*_(5,155)_ = 3.18, *p* = 0.009), as well as a significant three-way interaction Time * Cluster * Condition (*F*_(25,775)_ = 2.27, *p* < 0.001). We performed separate ANOVAs for each time window, with factors Cluster (all clusters) and Condition (ORD vs. RDM; see Figure 3A). In the 300–600 ms time window we observed main effects for Cluster (*F*_(5,155)_ = 3.79, *p* = 0.003), Condition (*F*_(1,31)_ = 5.55, *p* = 0.025) and a significant interaction Cluster * Condition (*F*_(5,155)_ = 5.25, *p* < 0.001). A similar result was obtained in the 600–900 ms time window, with a main effect for Cluster (*F*_(5,155)_ = 2.98, *p* = 0.014) and a significant interaction Cluster * Condition (*F*_(5,155)_ = 4.61, *p* = 0.001). No other time windows revealed significant interactions Cluster * Condition (*p*s > 0.1). Pairwise *t*-tests in 300–600 ms and 600–900 ms time windows revealed significant differences across conditions in the parieto-occipital left (*t*_(31)_ = 2.6, *p* = 0.014), parieto-occipital right (*t*_(31)_ = 3.26, *p* = 0.003) for the 300–600 ms time window, and in the parieto-occipital right cluster (*t*_(31)_ = 2.32, *p* = 0.027) for the 600–900 ms time window (see Figures 3B,C).

#### Gamma Band

**Figure 4:**
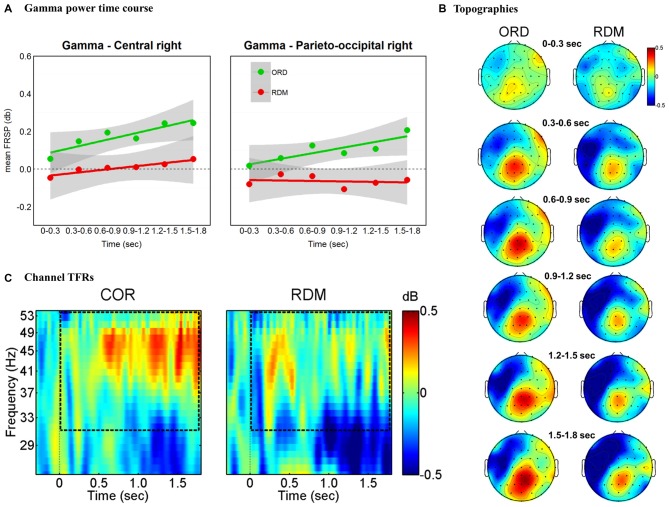
**Gamma band (31–55 Hz) effects time-locked to sentence onsets. (A)** Time course of gamma power for right parieto-occipital and central clusters. Mean power values are plotted in successive 300 ms time windows, up to 1800 ms. We fitted a linear regression line for each condition (green and red lines). The shaded gray regions represent a pointwise 95% confidence interval. **(B)** Topographic maps of gamma (31–55 Hz) power change in successive 300 ms time windows, up to 1800 ms. **(C)** TF representations of power changes at the sentence-level analysis. The black rectangles indicate the frequency range of interest. Results are plotted at one representative channel (PO4).

The findings for the gamma band are illustrated in Figure 4. The overall ANOVA revealed main effects for Cluster (*F*_(5,155)_ = 13.6, *p* = 0.000), Condition (*F*_(5,155)_ = 11.2, *p* = 0.002) and significant interaction Time * Cluster * Condition (*F*_(25,775)_ = 1.76, *p* = 0.013). A separate ANOVA for each time window with factors Cluster (all clusters) and Condition (ORD vs. RDM) revealed a significant main effect for Condition in all time windows except the initial one (0–300 ms). In order to further investigate the time course of power in the gamma band we followed a procedure similar to the one applied by Bastiaansen et al. (2010). We extracted average power change values in each cluster and we fitted a linear regression line for each individual subject (see Figure 4A). Hence, for each condition, we performed a one-sample *t*-test on individual beta weights—testing whether the regression slopes were significantly greater than zero (i.e., one-sided test). Results showed a linear trend in the ORD condition at parieto-occipital and central right clusters (*t*_(31)_ = 1.74, *p* = 0.045 and *t*_(31)_ = 1.7, *p* = 0.049, respectively) but not in the RDM condition for the same clusters (*t*s < 1; see Figures 4B,C). Pairwise* t*-tests comparing beta weights between conditions, revealed significantly larger regression coefficients for the ORD than the RDM condition in the right parieto-occipital cluster (*t*_(31)_ = 2.73, *p* = 0.01) but not in the right central cluster (*t*_(31)_ = 1.19, *p* = 0.24).

## Discussion

The main objectives of the present study were to investigate oscillatory brain dynamics during self-paced sentence-level reading and compare our fixation-related results with previous SVP findings. Although several different frequency bands were previously associated to language related functions (for reviews see Bastiaansen and Hagoort, 2006; Lewis et al., 2015), we focused on beta and gamma band modulation as predicted by Lewis and Bastiaansen’s (2015) framework for neural dynamics during sentence-level language comprehension. We used an adaptation of Bastiaansen et al.’s (2010) paradigm and asked participants to silently read semantically correct sentences, sentences containing a semantically unrelated word (henceforth: target word) and “sentences” in which the order of the words was randomized (no syntactic structure left). At the target word level, words that were both syntactically and semantically congruous with the sentential context showed typical eye-movement measures for self-paced reading (i.e., FFDs of *M* ~ 200 ms, GDs of *M* ~ 230 ms and TVTs of *M* ~ 240 ms—c.f., Rayner, 1998; Sereno and Rayner, 2003). Expectedly, semantically unrelated words embedded in otherwise correct sentences led to longer TVT (*M* = 270 ms) due to more regressive refixations after the first encounter of the word (i.e., during second-pass reading).

For the analysis of oscillatory brain dynamics we investigated power changes time-locked to the onset of the first fixation on the target word (target word analysis) and traced the evolution of power in the theta and gamma bands during the syntactic parsing of the sentence (sentence-level analysis). The target word analysis focused on the comparison between the two conditions with preserved syntactic structure (i.e., fully correct sentences vs. sentences containing a semantic violation). Larger beta band (13–18 Hz) desynchronization was found when participants read semantically incongruent words as compared to congruent words. Sentence-level analysis focused on comparing the conditions with a correct syntactic structure vs. the “sentences” with a random word order. On the sentence level, gamma power (31–55 Hz) increased linearly during parsing of syntactically correct sentences, whereas this effect was not observed when the order of the words was randomized. Furthermore, in the 300–900 ms time window after sentence onset, theta power (4–7 Hz) was greater for syntactically correct sentences than for the random word sequences.

### Semantic Violations Induced a Lower-Beta band Desynchronization

At the target word level our results confirmed the expected modulation of beta band activity. The expectation—derived from previous studies—was a beta band desynchronization time-locked to syntactically and semantically unrelated words—previously reported by Weiss et al. (2005), Davidson and Indefrey (2007), Luo et al. (2010) and Meyer et al. (2013). In these studies, semantic violations elicited a beta suppression as early as 200 ms after stimulus onsets (Luo et al., 2010; Wang et al., 2012a) and the localization of the effect was over areas typically associated with the reading network (for a review see Price, 2012; Taylor et al., 2013; Martin et al., 2015). These locations included bilateral parietal and occipital sites as well as left temporal and inferior frontal regions (Wang et al., 2012a; Kielar et al., 2014, 2015). Accordingly, we found that semantically unrelated words induced an early (0–300 ms after fixation onsets) lower-beta band desynchronization—mainly distributed over left parietal and occipital sites.

Based on the work by Herrmann et al. (2004), we expected—concomitant with the beta band desynchronization—that congruent words would generate a larger gamma band synchronization than semantically incongruent words. Our results did not fulfill this expectation. However, it is important to note that findings reported in the literature are inconsistent. On the one hand, several studies did not report gamma band effects for semantic violations (Hagoort et al., 2004; Davidson and Indefrey, 2007; Wang et al., 2012a; Kielar et al., 2014, 2015). On the other hand, numerous studies reported significant gamma band modulation (Hald et al., 2006; Penolazzi et al., 2009; Wang et al., 2012b; Rommers et al., 2013). Lewis and Bastiaansen (2015) interpreted these discrepancies in the literature to reflect differences due to different presentation modalities (i.e., single word presentations, sentence-level language comprehension, speech processing). In our FRSP analysis we used a 1000 ms baseline previous to the onset of the fixation on the target word. Thus, during the baseline, the reading process was already ongoing. This is not the case in SVP paradigms, where the inter-stimulus interval (i.e., a blank screen) is usually used as baseline period. The different cognitive affordances of the chosen baseline periods are a potential explanation for the divergent results when we compare findings stemming from different presentation modalities. However, of main interest for our target word analysis were changes in the ongoing sentence-level oscillatory dynamics during natural reading of continuous sentences (realized by the fixation-related setup). Using a blank screen as baseline period would create different cognitive affordances in the pre- and post-fixation onsets intervals—probably influencing the results.

Importantly, at the target word level, our beta and gamma effects are inconsistent with those from a previous fixation-related study (Metzner et al., 2015), whereas we share crucial commonalities with previous SVP findings (Kielar et al., 2014, 2015). Metzner et al.’s (2015) divergent findings (with regard to the original findings of Hagoort et al., 2004) led to the conclusion that oscillatory brain dynamics are qualitatively different when one compares self-paced reading with serial-visual presentation. In the present study, at the target word level, the observed pattern of results is in agreement with predictions of Lewis and Bastiaansen’s (2015) framework (which is, to a great extent—based on the work of Hagoort et al., 2004). This is noteworthy since this framework is primarily based on findings from SVP. Accordingly, we interpret the beta band desynchronization to reflect the revision/change of the ongoing NCN prompted by semantically unrelated words (see also Engel and Fries, 2010; Bressler and Richter, 2015). Furthermore, we expected semantic violations to generate gamma band desynchronization resulting from the mismatch between top-down sentence-level predictions and bottom-up incoming semantic violations (Herrmann et al., 2004). The gamma band effect did not reach significance at the target word level, however support for this hypothesis can be found in our sentence-level analysis.

### Theta and Gamma Power During Syntactic Parsing of the Whole Sentences

For the sentence-level analysis Bastiaansen et al. (2010) reported a linear increase in power of lower beta (13–18 Hz) and theta (4–8 Hz) bands for syntactically correct sentences. The authors did not observe such an effect when the order of the words was randomized. In the present study, sentence-level analysis did not reveal significant differences in beta band power when we compared fully correct sentences to “sentences” with a random word order. According to Lewis and Bastiaansen’s (2015) framework, an increase in beta activity is to be expected under the condition in which supplementary load is added to the current NCN. NCN’s load has been associated to beta band activity during experiments investigating syntactic unification processes (Weiss et al., 2005; Bastiaansen and Hagoort, 2006) and in experiments investigating subject-verb agreement dependencies (Meyer et al., 2013). As aforementioned, in SVP paradigms the pace at which words are presented is externally controlled. Presentation rates in the range of 500–1000 ms per word may lead to processing differences when compared to the average 200–250 ms fixation duration in natural reading (Rayner, 1998; Pernet et al., 2007; Kliegl et al., 2012). An externally controlled (i.e., superimposed) timeline of visual word recognition is likely to call for additional processing load on the NCN which may be reflected in the beta increase observed by Bastiaansen et al. (2010). On the contrary, in self-paced reading paradigms, subjects are free to read at their own pace without any further restrictions above and beyond visual word recognition. Thus, differences in the NCNs’ processing load, imposed by manners of presentation, might have led to the pattern of results observed in the present study.

Additional support for the interpretation that the divergent findings are due to differences in the processing load of the NCN can be found in speech processing experiments. Peña and Melloni (2012) had participants listen to utterances played in their native language and utterances played in foreign languages. Both native and foreign languages were played either forward or backwards. As in our self-paced reading experiment, the authors did not find a linear increase in beta band power for utterances played forward and in the native language. Results in the beta band are inconsistent when we consider similar experimental conditions, but different manners of presentation (i.e., SVP, self-paced reading, spoken sentence processing). Task-related effects on beta band modulation will require further empirical investigation. Besides no beta effects, Peña and Melloni (2012) reported a significant increase in theta and gamma band power when participants listened to utterances played forward and in their native language. In the present experiment, we observed a linear increase in gamma power at the posterior right site when participants read syntactically correct sentences. For the same site, this trend was not observed when the order of the words was randomized. We interpret the gamma modulation as reflecting the successful sequential matching of top-down sentence-level predictions and bottom-up information (Herrmann et al., 2004; Lewis and Bastiaansen, 2015).

It could be argued that the observed gamma modulation reflects the sequential encoding of words into a multi-item working memory system. This hypothesis would be in line with studies reporting a linear increase in gamma power as a function of increasing working memory load (Howard et al., 2003). However, we consider this to be an unlikely explanation, as no such a trend was observed in previous SVP experiments (where the memory load is greater compared to natural reading paradigms; Kliegl et al., 2012) or when the order of the words was randomized. In previous sentence-level SVP studies (see Bastiaansen et al., 2010) not gamma, but theta power linearly increased across the sentences. Theta band modulation was associated with memory and unification processes (Hald et al., 2006; Meyer et al., 2015) as well as retrieval of specific items from a working memory set (Bastiaansen and Hagoort, 2006). We observed larger theta power for syntactically correct sentences compared to the random words condition—restricted to the 300–900 ms time window after sentence onset. Again, these findings could stem from the different working memory loads imposed by manners of presentation. In Bastiaansen et al. (2010), sentence presentation lasted up to 6 s, whereas in our case the average time needed by participants to complete sentences was just above 2 s. Unification and retrieval of specific items from a working memory set might be more challenging when words constituting a sentence are presented one by one and separated by long ITIs (Raghavachari et al., 2001).

## Conclusions

The pattern of results evidenced by the present study is in line with the predictive coding framework for rapid oscillatory neuronal dynamics described by Lewis and Bastiaansen (2015). Unlike Metzner et al.’s (2015) results, our fixation-related analysis revealed qualitatively comparable findings as the SVP literature. Despite several shared commonalities, presentation modality was identified as the most likely explanation for the observed differences in the temporal distribution of the effects.

For the present study, the analysis of power measures was performed on the basis of pre-defined clusters of electrodes (see “Time-frequency analysis” Section). However, it should be noted that more sophisticated data-driven clustering methods, such as Threshold Free Cluster Enhancement (TFCE, Smith and Nichols, 2009; Ehinger et al., 2015; Pernet et al., 2015) and cluster-based permutation test (Maris and Oostenveld, 2007) would provide a valuable alternative to literature-based clusters. One additional challenge associated to the fixation-related approach, was the correction of EM related artifacts. For the present study we adopted a classical approach (Jung et al., 1998, 2000) which proved to be sufficiently accurate in detecting and removing EM related activity. However, more advanced methods for the identification, characterization and correction of EM artifacts, could further improve the quality of the signal, hence the stability of the results in future studies (see Dimigen et al., 2011; Plöchl et al., 2012). Last, methodological choices (e.g., the baseline period, the experimental design and the preferred power analysis method) are well established sources of heterogeneity in the field and issues for future research.

To date, SVP paradigms were the most extensively used tool to investigate word recognition in sentential context (for a review see Bastiaansen and Hagoort, 2006). SVP paradigms were, however, criticized for the restrictions imposed on reading-related EM behavior (e.g., word skippings, regressive saccades) and reading-related attentional processes (e.g., parafoveal preprocessing; Sereno and Rayner, 2003; Schotter et al., 2012). FRPs and FRSPs were proven to be reliable tools in the study of neural underpinnings of the reading process. We hope that future research will benefit from these newly pioneered techniques by studying reading and reading related processes from a more ecologically valid perspective.

## Author Contributions

Conceptualized and designed the experiment (LV, FH, SH); acquired the data (LV, NAH); analyzed the data (LV, FR); wrote the article (LV, NAH, FH, SH, FR). All authors have approved the final version of the article and agree to be accountable for all aspects of this work.

## Conflict of Interest Statement

The authors declare that the research was conducted in the absence of any commercial or financial relationships that could be construed as a potential conflict of interest.
